# Recent developments in the catalytic conversion of cellulose

**DOI:** 10.1080/13102818.2014.980049

**Published:** 2014-11-14

**Authors:** Yan Wang, Hang Song, Lincai Peng, Qiangsheng Zhang, Shun Yao

**Affiliations:** ^a^ Department of Pharmaceutical and Biological Engineering, College of Chemical Engineering, Sichuan University, Chengdu, Sichuan, P. R. China

**Keywords:** cellulose, conversion, hydrolysis, gasification, pyrolysis

## Abstract

The increasing demand for energy has led to the development of biomass conversion technologies. As the most abundant biomass on Earth, cellulose is generally chosen as the primary research target for biomass conversion. In this review, gasification and pyrolysis of cellulose are briefly discussed and hydrolysis is then considered in detail. Moreover, many new developments and applications are introduced in cellulose conversion in recent years. Among these technologies, heterogeneous catalysis, hydrolysis in ionic liquid and hydrolysis by hot-compressed water exhibit a promising potential in cellulose conversion. Therefore, they are well recognized as powerful, fast and efficient techniques, becoming the focus of intensive research.

## Introduction

**Figure 1.**
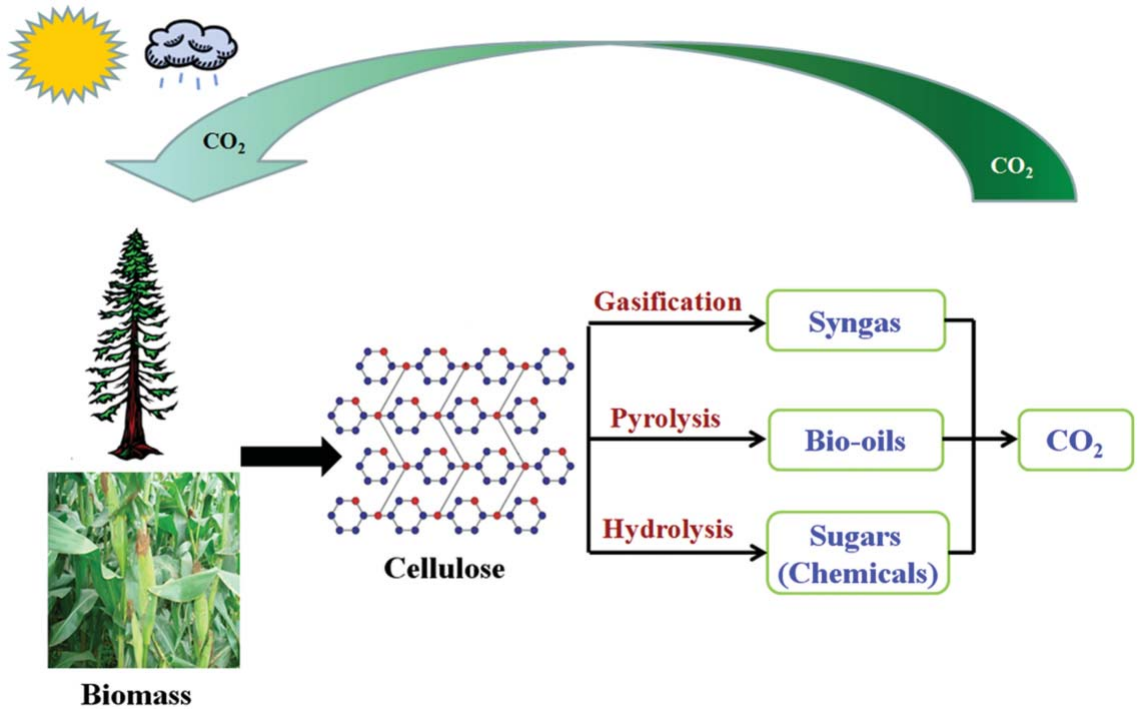
Integrated biomass-energy-chemicals for sustainable technologies.

In recent years, considerable attention is being paid to the gradual depletion of fossil fuel reserves in the near future. In order to meet the ever-growing energy demands and environmental concerns, increasing research efforts have been devoted to seeking substitutes for energy and chemical production. Compared to traditional energy sources, such as petroleum, coal and natural gas, lignocellulosic biomass is abundant, inexpensive and renewable. It has great potential to solve two issues: the global energy crisis and the greenhouse effect (Figure 1). Therefore, it is a promising substitute of the fossil-based feedstock.[1]

Among the three main components in lignocelluloses (cellulose, hemicellulose and lignin), cellulose, which is with a content of 40%–50% in lignocellulose, is known to be the most abundant biopolymer in nature.[2] Cellulose, which is composed of β-1,4-glycosidic bonds of D-glucose, has a highly ordered crystal structure owing to the hydrogen-bond network between hydroxyl groups. As a result, it has difficult degradation and is insoluble in conventional solvents, such as water. Thus, the efficient conversion of cellulose into fuels and valuable chemicals plays an important role in the production of sustainable energy.

Up to now, there have been three main methods to convert cellulose. Gasification has been used to produce syngas for methanol and Fischer–Tropsch synthesis. Pyrolysis or liquefaction leads to production of complexed liquid fuels. As to hydrolysis, three well-known catalysts (homogeneous acids, solid catalysts and enzymes) have been utilized to hydrolyze cellulose. This method receives intensive interest on both academic and industrial levels. Moreover, a large number of applications of several combinations of reactions (hydrolysis–hydrogenation, hydrolysis–oxidation, etc.) have been successively reported in cellulose conversion.

In this review, we describe the recent advances in the conversion of cellulose. Particular emphasis will be placed on hydrolysis of cellulose into valuable platform compounds. First, we illustrate the cellulose reactivity in the presence of homogeneous acid catalysts, heterogeneous catalysts and enzymes, respectively. Then, we describe the uses of ionic liquids and water in supercritical or subcritical states. Although intensive researches have been carried out in the field of cellulose conversion, the direction of cellulose transformation has been towards improving the obtained products and making the process more environmentally friendly. The drawbacks of previous processes in cellulose transformation favour the development of novel pathways.

## Gasification of cellulose

From the point of view of environmental protection, biomass gasification provides some considerable advantages. It is a possible alternative energy source. Since the mid-1980s, growing interest has been drawn on the subject of catalytic biomass gasification.[3] Intensive investigations have been conducted to discover more efficient processes for biomass gasification to syngas.[4,5] Asadullah et al. [6] developed an efficient process for cellulose gasification at low temperature in a continuous feeding fluidized bed reactor. An overview of available reports shows that research on catalysts for use in the process is often carried out specifically.

### Dolomite catalysts

Dolomite, a mineral with the general chemical formula CaMg(CO_3_)_2_, has attracted much attention in catalytic gasification of biomass. Calcined dolomite (MgO–CaO), MgO and CaO have been evaluated for the steam reforming of biomass tars.[7] Moreover, the effects of temperature, contact time and the catalyst particle diameter have been investigated, as well as the tar conversion, tar concentration in the exit gas, gas yield and gas composition. The results showed that the order of activity was dolomite > magnesite > calcite. An experiment [8] on the effect of four different dolomites in Spain (Chilches, Malaga, Norte and Sevilla) showed that the order of activity was Norte > Chilches > Malaga > Sevilla.(reviewed in [3]) Interestingly, the authors revealed that the higher activity of the Norte and Chilches dolomites may be attributed to their higher Fe_2_O_3_ content and larger pore diameters.

### Ni-based catalysts

The majority of the literature available on biomass gasification concerns Ni-based catalysts. Using these catalysts, there is universally an increase in the hydrogen and carbon monoxide content in the exiting gas, with a reduction of the hydrocarbon and methane content.[3] Rapagna et al. [9] performed catalytic biomass steam gasification runs in a fluidized bed gasifier, followed by a catalytic fixed-bed reactor. The effect of the operating conditions in the catalytic converter on the distribution of gases, especially H_2_, showed H_2_ content of over 60% by volume. Moreover, Ni-based catalysts were demonstrated to be extremely active in eliminating CH_4_ and tars.

Although biomass gasification technology has made great progress, there are still certain limitations. Further research is needed in the following aspects: (1) to seek highly active, stable, reproducible and inexpensive catalysts. (2) To improve processes for achieving high efficiency, low energy consumption and comprehensive utilization of biomass energy. (3) To design a novel gasifier.

### Pyrolysis of cellulose

In recent years, a great deal of attention has been drawn on the thermal degradation of cellulose. There has been considerable controversy over the mechanism and kinetics of the decomposition reactions in the past few decades.[10] Generally, cellulose pyrolysis can be divided into the following types: slow pyrolysis, fast pyrolysis, flash pyrolysis and catalytic pyrolysis.

Piskorz et al. [10] showed that conversion of cellulose to anhydro-oligomers could be achieved by flash pyrolysis. These compounds were obtained in a yield of up to 20% of the cellulose fed. In another experiment,[11] the authors demonstrated that decomposition of cellulose to levoglucosan (anhydroglucose) could be realized by fast pyrolysis in a fluidized bed. Large-scale microwave rapid pyrolysis of cellulosic materials was investigated by Miura et al. [12], who examined the effects of material sources on the levoglucosan yield. The result suggested that the amount of the obtained levoglucosan is proportional to the content of cellulose, that is, a higher content of cellulose gives a larger amount of levoglucosan.[12]

Intensive investigations have been conducted to discover more efficient processes for biomass pyrolysis to bio-oils. However, a few questions still remain. For example, since bio-oils are complex and chemically unstable mixtures, much more work needs to be done on their stabilization and upgrading. The equipment configuration should be modified before the actual application of bio-oils.[13] These problems are also the bottleneck of cellulose pyrolysis in the industrial processes. Therefore, further research needs to be done.

### Hydrolysis of cellulose

Efficient disruption of the hydrogen-bond network and hydrolytic cleavage of the β-1,4-glycosidic bonds in cellulose fibres plays an essential role in cellulose degradation. Generally, cellulose depolymerization can be divided into two steps: cellulose selectively hydrolyzed into sugars, followed by further conversion into fuels and chemicals. So far, the hydrolysis of cellulose has got a remarkable progress. Many methods have been developed for decomposition of cellulose, such as acidic/alkaline hydrolysis, enzymatic hydrolysis and hydrolysis in ionic liquid. The characteristics of these transformed methods are summarized in Table 1. Meanwhile, some researchers have committed themselves to develop a single-step catalytic process for conversion of cellulose into fuels or chemicals.

**Table 1.** Characterization of the methods for decomposition of cellulose.

Method	Advantages	Disadvantages
Homogeneous hydrolysis	(1) Less expensive, i.e., conventional inorganic acid can be used; (2) Achieving high activity.	(1) Energy inefficient. High temperatures are always required; (2) Equipment corrosion is easy to cause; (3) Inconvenient for separation and recycling. The method produces large amounts of waste water.
		
Heterogeneous hydrolysis	(1) Convenient for separation and recyclability; (2) A wide variety of solid catalysts are available; (3) Environmentally friendly; (4) High hydrothermal stability.	(1) High catalyst/substrate mass ratio is often needed; (2) The transfer resistance between catalysts and insoluble or partially soluble cellulose restricts the catalytic activity.
		
Enzymatic hydrolysis	(1) Highly selective; (2) Mild reaction conditions.	(1) Many enzymes are costly; (2) Sensitive to operating conditions, such as pH, temperature and presence of some inhibitors; (3) A small number of successful recovery methods are available.
		
Ionic liquid hydrolysis	(1) Flexible because various ionic liquids can be synthesized; (2) Good performance in dissolving cellulose; (3) Effective recovery and recycling can be achieved.	(1) Many ionic liquids are expensive; (2) The recovery of the ionic liquid is a highly energy-consuming process.

#### Acid-catalyzed hydrolysis of cellulose

According to the currently available literature, acid-catalyzed cellulose transformation can be roughly divided into liquid acid transformation and solid acid transformation. Both of the two catalyzed methods have their advantages and disadvantages.

##### Acid homogeneous catalysis for cellulose hydrolysis

Since the fact that sugars are decomposed under severe conditions needed to facilitate cellulose hydrolysis, i.e., high temperature and low pH, in recent years there is a decreasing trend in the interest in acid technology.[14] Nevertheless, there are still constant research efforts on the subject and a significant development has been made in the area of homogeneous acid hydrolysis of cellulose. Dilute acid processes were industrialized in the early part of the 20th century.[15]

Torget et al. [16] proposed a homogeneous kinetic model for cellulose hydrolysis. The results showed that the initial cellulose hydrolysis rate constant using sulphuric acid (0.07 wt%) in a flowing percolation reactor is enhanced five-fold, compared with a batch reactor. In addition, using relatively mild hydrolysis severities, biphasic catalysis was observed. Another experiment [17] gave nearly 50% glucose yield from cellulose under the operating conditions of 230 °C, 15 s residence time and 1% acid. In this work, a surprising observation was made that the decomposition of cellulose is basically acidity dependent: the more acidic the homogeneous catalyst, the better the yields of glucose. This phenomenon was also observed in another similar study.[18]

Although liquid acids are highly active in the process of cellulose hydrolysis, the process suffers from energy inefficiency, corrosion hazard, difficulties in the separation and recycling of acids, as well as production of large amounts of waste. Considering these disadvantages, it is urgently necessary to develop a more sustainable approach.

##### Cellulose transformation by heterogeneous catalysis

In order to avoid the limitations of homogeneous catalysis, a new approach, based on the use of heterogeneous catalysts, emerged in 2006.[19] Since solid acids can be easily separated and recycled from the liquid reaction system, in the following years, much effort was done in the field of heterogeneous catalysts.

Onda et al. [20] described that sulphonated activated carbon (AC-SO_3_H) provided excellent catalytic properties for the hydrolysis of cellulose. The authors discovered that the AC-SO_3_H catalyst gave 41% glucose yield and had 95% selectivity at moderate temperature (150 °C), while a higher temperature induced degradation of the produced glucose. Moreover, the H-form zeolite catalysts and the sulphated catalysts were tested for comparison with the sulphonated activated carbon. The resultant high yield of glucose in sulphonated activated carbon accounted for the strong acid sites of SO_3_H functional groups and the hydrophobic planes. Furthermore, Pang et al. [21] improved the glucose yield to 75% with 94% conversion of cellulose on sulphonated mesoporous carbon (CMK-3). Recently, Wang et al. [22] reported a series of solid acids for the hydrolysis of cellulose in the 1-butyl-3-methylimidazolium chloride ([BMIM]Cl) under microwave irradiation to produce 5-hydroxymethylfurfural (5-HMF). Interestingly, it was found that the total reducing sugars (TRS) yields with solid acids were higher than those with chromium, whereas HMF yields were lower than those catalyzed by chromium.

Other successful catalysts have been evaluated in succession. The performance of several solid acid catalysts for cellulose conversion is summarized in Table 2.

**Table 2.** Hydrolytic degradation of cellulose with solid acid catalysts.

Entry	Catalyst	Cellulose	Conversion (%)	Yield of glucose (%)	Ref.
(1)	Cs_2_HPW_12_O_40_	Avicel	31.5	5^a^	[23]
(2)	Tungstated zirconia	Avicel	42	19^a^	[23]
(3)	Dowex 50WX8	Avicel/Wood	n.r.	17.2 / 11.5	[24]
(4)	Nafion SAC 13	Cellobiose	11	9(2^b^)	[25]
(5)	Silica/carbon nanocomposites	Avicel	60.7	50.4	[26]

Note: a,b – the product is lactic acid or levulinic acid, respectively.

n.r. – not reported.

Ref. – reference.

Heterogeneous catalysis for cellulose conversion has attracted much attention. However, the fact that transfer resistance between solid acid and insoluble, or partially soluble, cellulose will restrict the catalytic activity should be taken into account.[27] Therefore, it is very important how to promote mass and heat transfer, in order to enhance the activity of given solid acid catalysts for glycosidic bond cleavage in cellulose. Up to now, two main options have been described: reducing cellulose crystallinity or optimizing reaction conditions. The development of this field is still in progress.

#### Cellulose degradation by alkalis

Unlike acid catalysts, the hydrolysis of cellulose in alkaline medium has got, more or less, certain development. Alkaline degradation includes end-wise degradation, termination, alkaline scission and oxidative alkaline degradation.[28] At temperatures below 170 °C, β-1,4-glycosidic linkages are relatively stable, yet a notable decrease in molecular weight is observed when cellulose is boiled with dilute sodium hydroxide at such temperature. When cellulose is treated at a higher temperature, formic, acetic and lactic acid can be generated as main chemicals with lower molecular weight. Nevertheless, cellulose degradation by alkalis cannot usually be considered as a selective route to produce glucose on the account of low yield. On the contrary, basic treatment is often used as a pretreatment to increase cellulose accessibility.[27]

In addition, some heterogeneous alkaline catalysts were tested for cellulose conversion in hydrothermal conditions and reductive atmosphere. For instance, Jollet et al. [29] examined the cellulose transformation by basic solid catalysts in aqueous phase. The result showed poor activities in cellulose conversion.

### Enzymatic hydrolysis of cellulose

The quantity of scientific research on enzymatic hydrolysis of cellulose for production of bio-ethanol and other value-added organic compounds has expanded dramatically in recent years. It received intensive attention on academic level, due to its highly selective nature under mild reaction conditions. It is well known that the concept of bio-refinery is emerging to replace an already existing petro-refinery as the latter is supposed to become exhausted in the near future.

Ethanol production from lignocellulosic biomass is one of the most important approaches for production of renewable transportation fuels. Until now, there has been an increased interest in commercializing technologies for bioethanol from inexpensive biomass.[30] Additionally, physical changes of cellulose, i.e., swelling, segmentation or destratification, play an important role in enhancement of enzymatic hydrolysis. The relationship between the structural properties of cellulose and the rate of enzymatic hydrolysis has been the subject of intensive research.[31] Several studies [31–33] have investigated the roles of crystallinity index, degree of polymerization and accessibility in impacting hydrolysis. It was found that cellulose hydrolysis rates mediated by fungal cellulases are typically 3–30 times faster for amorphous cellulose as compared with high crystalline cellulose.[31] In the meantime, cellulase systems have been comprehensively reviewed [31,34] during the past three decades.

The enzymatic hydrolysis process opens up new opportunities for efficient use of cellulose. However, one of the remaining obstacles in such catalytic reactions is yet to be overcome. Namely, enzymes are sensitive to operating conditions, such as pH, temperature and presence of some inhibitors. Moreover, the high prices also suppress intensive industrialization. Clearly, further investigations in developing more stable and cheap enzymes are required.

### Hydrolysis in ionic liquids

Over the past several years, ionic liquids, due to their fascinating properties, such as high thermal stability, negligible vapour pressure, wide liquid temperature range and tunable solubility, have received considerable interest. The application of ionic liquids as solvents for the dissolution of cellulose was initially reported by Swatloski et al.[35] This discovery opened up a new pathway to deal with cellulose at low temperatures. Subsequently, hydrolysis of cellulose into sugars and valuable chemicals was observed when liquid acid or solid acid was added to the reaction system with ionic liquids as solvents.[36,37] Furthermore, systems in which an ionic liquid not only serves as a solvent, but also as a catalyst, have attracted some attention in cellulose hydrolysis. In currently emerging investigations, cellulose transformation in ionic liquid can be roughly divided into the following two systems: Lewis acid systems and Brønsted acid systems.

#### Hydrolysis in Lewis acid ionic liquids

Since ionic liquids have been used to dissolve cellulose, an increasing number of studies are being carried out to investigate their catalysis on cellulose conversion. Su et al. [38] achieved a single-step conversion of cellulose to 5-HMF with a pair of metal chlorides (CuCl_2_ and CrCl_2_) as catalysts in 1-ethyl-3-methylimidazolium chloride ([EMIM]Cl) solvent. The authors demonstrated that the rate of cellulose depolymerization was about one order of magnitude faster than that in conventional acid-catalyzed hydrolysis. More interestingly, single metal chloride, at the same total loading, showed considerably less activity under similar conditions. This interesting phenomenon was also observed in another experiment.[39] Therefore, a detailed mechanism for the notably higher activity in the presence of paired metal chlorides remains to be further unveiled. In addition, a study launched by Hsu et al. [40] investigated the effect of H_2_O/cellulose molar ratio, temperature, time and ionic-liquid type on the production of monosaccharides and 5-HMF in [EMIM]Cl and *N*-ethylpyridinium chloride ([Epyr]Cl). Besides, Li et al. [41] observed the acidolysis of three wood species in the ionic liquid 1-allyl-3-methylimidazolium chloride in the presence of small amounts of hydrochloric acid. The authors observed that aqueous reactions (under identical acid concentrations) showed a remarkably lower efficiency. The result demonstrated that ionic liquids offer a unique environment for acid-catalyzed dehydration chemistry.

Collectively, the catalytic methods in the group of Lewis acid systems are generally a combination of ionic liquids (R^+^ X^−^) and metal chlorides (MX*_n_*), which typically results in the formation of RMX*_n_*_+1_ (mononuclear) or RM*_m_*X*_mn_*_+1_ (polynuclear).[38] However, more attention should be paid on the detailed mechanism of the catalytic activity of Lewis acid systems in cellulose conversion.

#### Hydrolysis in Brønsted acid ionic liquids

It is urgent to find more efficient systems because of the relatively lower activity in Lewis acid systems. Lately, there has been growing interest in Brønsted acid ionic liquid. Recently, Tao et al. [42] reported the transformation of cellulose to 5-HMF and furfural with high yields in the presence of 1-(4-sulphonic acid)-butyl-3-methylimidazolium hydrogen sulphate. They emphasized that functional acidic ionic liquid was an effective catalyst for the hydrolysis of cellulose. Shortly afterwards, the group investigated the effect of acidity and structure of SO_3_H-functionalized ionic liquids on cellulose hydrolysis.[43] Amarasekara and Wiredu [44] also focused on the conversion of cellulose into TRS in ionic liquid 1-(1-propylsulphonic)-3-methylimidazolium chloride solutions. When comparing with *p*-toluenesulphonic acid and sulphuric acid of the same acid strength, 28.5%, 32.6% and 22.0% yields of TRS were obtained, respectively, after heating at 170 °C for 3 h.

Additionally, Liu et al. [45] investigated six kinds of SO_3_H-functionalized acidic ionic liquids based on 1-methylimidazole, 1-vinylimidazole and triethylamine, which were applied as catalysts to promote the hydrolysis of cellulose in [BMIM]Cl. The result showed that triethyl-(3-sulpho-propyl)-ammonium hydrogen sulphate was the most appropriate ionic liquid for cellulose hydrolysis, with a maximum TRS yield of over 99%. Moreover, it demonstrated that the water in [BMIM]Cl had a conspicuous effect on cellulose hydrolysis. A decrease of TRS yield from 99% to 27.9% was observed when 0.0261 g of H_2_O was added in [BMIM]Cl. Therefore, controlling the amount of water at a comparatively low level is a critical issue.

It is well known that Brønsted acids normally perform at stronger acidity than Lewis acids. Most research indicates that acidity plays a crucial role in cellulose hydrolysis. Recently, some novel investigations in which a Lewis acid ionic liquid acts as a solvent, while a Brønsted acid ionic liquid serves as catalyst, were conducted.[45] The concept of difunctional ionic liquids is emerging to replace already existing catalytic systems. Moreover, if this kind of difunctional ionic liquid is temperature sensitive, its separation from the reaction system will be simply achieved after cooling down. Thus, exploitation of this novel type of ionic liquids may gain wide popularity in future.

### Other catalytic systems

Since some researchers have proposed that esters undergo rapid hydrolysis in near-critical or in supercritical water without any catalyst,[46] the hydrolysis of cellulose in near-critical or supercritical water received intensive attention. In [47] and [48], the hydrolysis behaviour in amorphous and crystalline portions of microcrystalline cellulose is compared. It was found that the glycosidic bonds in the amorphous portion started to hydrolyze into glucose monomers from around 150 °C, while for the crystalline part, the process of hydrolysis started at about 180 °C. This may be attributed to the hydrogen bonds in the crystalline part, thus, excessive energy was required for the hydrolysis of the crystalline part. Very recently, microwave irradiation was introduced into cellulose hydrolysis systems. The promoting effect of microwave irradiation on hydrolysis of cellulose has long been recognized. Recently, Yu and Wu [47] and Wu et al. [48] reported the solid acid-catalyzed hydrolysis of cellulose in water under microwave irradiation. As a result, the system gave a yield of 24% reducing sugars at 90 °C after 60 min.

Thus, as mentioned above, several combinations of reactions (hydrolysis–hydrogenation, hydrolysis–oxidation, etc.) were successively applied in cellulose conversion. Geboers et al. [49], for example, investigated the conversion of ball-milled cellulose in the presence of heteropoly acids (H_3_PW_12_O_40_ and H_4_SiW_12_O_40_) and commercial Ru/C catalysts. A hexitol yield of 85% was obtained at 190 °C and 6 MPa of H_2_ pressure within one hour. Tan et al. [50] reported one-pot oxidation of cellobiose into gluconic acid over Au/CNTs (CNT, carbon nano tube). The catalytic process gave a 68% yield of gluconic acid at 81% cellobiose conversion. Although this example refers only to oxidation of cellobiose, similar catalytic behaviour would be conceivable starting from cellulose with reduced crystallinity.

## Conclusions

A major trend in conversion of cellulose is clearly heading towards faster and more efficient conversion with comparable or improved conversion capability. After so many years, gasification and pyrolysis, as well as hydrolysis, have become an indispensable part of cellulose transformation. Particularly, the hydrolysis of cellulose has received extensive attention. Unfortunately, severe mass-transfer limitations hamper the progress in the processing of hydrolysis. Recent developments, such as introduction of microwave-assisted protocols, use of ionic liquids for cellulose dissolution and depolymerization and biphasic reactor systems, can address these problems to improve the reactivity. However, since many of these catalytic processes are still in their infancy, further optimization of reaction conditions in order to attain higher product selectivity and greater tolerance of the catalysts towards dissolution and leaching, is essential for the future. Moreover, difunctional and temperature-sensitive ionic liquids are another promising catalytic methodology.

Collectively, more and more new techniques with high selectivity appear in the field of cellulose transformation. These advances will lead to persistent improvement of conversion capability.

## References

[cit0001] Corma A, Iborra S, Velty A (2007). Chemical routes for the transformation of biomass into chemicals. Chem Rev..

[cit0002] Wang Y, Song H, Hou JP, Jia CM, Yao S (2013). Systematic isolation and utilization of lignocellulosic components from sugarcane bagasse. Separation Sci Technol..

[cit0003] Sutton D, Kelleher B, Ross JRH (2001). Review of literature on catalysts for biomass gasification. Fuel Process Technol..

[cit0004] De Bari ID, Barisano D, Cardinale M, Matera D, Nanna F, Viggiano D (2000). Air gasification of biomass in a downdraft fixed bed: a comparative study of the inorganic and organic products distribution. Energy Fuels..

[cit0005] Di Blasi C, Signorelli G, Portoricco G (1999). Countercurrent fixed-bed gasification of biomass at laboratory scale. Ind Eng Chem Res..

[cit0006] Asadullah M, Miyazawa T, Ito S, Kunimori K, Tomishige K (2003). Catalyst development for low temperature gasification of biomass: function of char removal in fluidized bed reactor. Stud Surf Sci Catal..

[cit0007] Delgado J, Aznar MP, Corella J (1997). Biomass gasification with steam in fluidized bed: effectiveness of CaO, MgO, and CaO-MgO for hot raw gas cleaning. Ind Eng Chem Res..

[cit0008] Orío A, Corella J, Narváez I, Bridgwater AV, Boocock DGB (1997). Characterization and activity of different dolomites for hot gas cleaning in biomass gasification. Developments in thermochemical biomass conversion.

[cit0009] Rapagna S, Jand N, Foscolo PU (1998). Catalytic gasification of biomass to produce hydrogen rich gas. Int J Hydrogen Energy..

[cit0010] Piskorz J, Majerski P, Radlein D, Vladars-Usas A, Scott  DS (2000). Flash pyrolysis of cellulose for production of anhydro-oligomers. J Anal Appl Pyrolysis..

[cit0011] Radlein D, Grinshpun A, Piskorz J, Scott DS (1987). On the presence of anhydro-oligosaccharides in the syrups from the fast pyrolysis of cellulose. J Anal Appl Pyrolysis..

[cit0012] Miura M, Kaga H, Yoshida T, Ando K (2001). Microwave pyrolysis of cellulosic materials for the production of anhydrosugars. J Wood Sci..

[cit0013] Zhang Q, Chang J, Wang TJ, Xu Y (2007). Review of biomass pyrolysis oil properties and upgrading research. Energy Conversion Manag..

[cit0014] Lee YY, Iyer P, Torget RW (1999). Dilute-acid hydrolysis of lignocellulosic biomass. Adv Biochem Eng Biotechnol..

[cit0015] Harris EE. Wood saccharification. In: Pigman WW, Wolfrom ML, editors. Advances in carbohydrate chemistry. New York: Academic press; 1949. p. 153–188.

[cit0016] Torget RW, Kim JS, Lee YY (2000). Fundamental aspects of dilute acid hydrolysis/fractionation kinetics of hardwood carbohydrates: cellulose hydrolysis. Ind Eng Chem Res..

[cit0017] Fan LT, Gharpuray MM, Lee YH (1987). Cellulose hydrolysis.

[cit0018] Baugh KD, McCarty PL (1988). Thermochemical pretreatment of lignocellulose to enhance methane fermentation: monosaccharide and furfurals hydrothermal decomposition and product formation rates. Biotechnol Bioeng.

[cit0019] Fukuoka A, Dhepe PL (2006). Catalytic conversion of cellulose into sugar alcohols. Angew Chem Int Edition..

[cit0020] Onda A, Ochi T, Yanagisawa K (2008). Selective hydrolysis of cellulose into glucose over solid acid catalysts. Green Chem..

[cit0021] Pang J, Wang A, Zheng M, Zhang T (2010). Hydrolysis of cellulose into glucose over carbons sulfonated at elevated temperatures. Chem Commun..

[cit0022] Wang P, Yu HB, Zhan SH, Wang SQ (2011). Catalytic hydrolysis of lignocellulosic biomass into 5-hydroxymethylfurfural in ionic liquid. Bioresour Technol..

[cit0023] Chambon F, Rataboul F, Pinel C, Cabiac A, Guillon E, Essayem N (2011). Cellulose hydrothermal conversion promoted by heterogeneous Brønsted and Lewis acids: remarkable efficiency of solid Lewis acids to produce lactic acid. Appl Catalysis B.

[cit0024] Watanabe H (2010). The study of factors influencing the depolymerisation of cellulose using a solid catalyst in ionic liquids. Carbohydr Polymers.

[cit0025] Hegner J, Pereira KC, DeBoef B, Lucht BL (2010). Conversion of cellulose to glucose and levulinic acid via solid-supported acid catalysis. Tetrahedron Lett..

[cit0026] Van de Vyver S, Peng L, Geboers J, Schepers H, de Clippel F, Gommes CJ, Goderis B, Jacobs PA, Sels BF (2010). Sulfonated silica/carbon nanocomposites as novel catalysts for hydrolysis of cellulose to glucose. Green Chem..

[cit0027] Cabiac A, Guillon E, Chambon F, Pinel C, Rataboul F, Essayem N (2011). Cellulose reactivity and glycosidic bond cleavage in aqueous phase by catalytic and non catalytic transformations. Appl Catalysis A.

[cit0028] Knill CJ, Kennedy JF (2003). Degradation of cellulose under alkaline conditions. Carbohydr Polymers..

[cit0029] Jollet V, Chambon F, Rataboul F, Cabiac A, Pinel C, Guillon E, Essayem N (2009). Non-catalyzed and Pt/γ-Al2O3-catalyzed hydrothermal cellulose dissolution–conversion: influence of the reaction parameters and analysis of the unreacted cellulose. Green Chem..

[cit0030] Schell DJ, Riley CJ, Dowe N, Farmer J, Ibsen KN, Ruth MF, Toon ST, Lumpkin RE (2004). A bioethanol process development unit: initial operating experiences and results with a corn fiber feedstock. Bioresour Technol..

[cit0031] Lynd LR, Weimer PJ, van Zyl WH, Pretorius IS (2002). Microbial cellulose utilization: fundamentals and biotechnology. Microbiol Mol Biol Rev..

[cit0032] Fierobe HP, Bayer EA, Tardif C, Czjzek M, Mechaly A, Bélaïch A, Lamed R, Shoha Y, Bélaïch JP (2002). Degradation of cellulose substrates by cellulosome chimeras substrate targeting versus proximity of enzyme components. J Biol Chem..

[cit0033] Mansfield SD, Mooney C, Saddler JN (1999). Substrate and enzyme characteristics that limit cellulose hydrolysis. Biotechnol Prog..

[cit0034] Zhang Y-HP, Lynd LR (2004). Toward an aggregated understanding of enzymatic hydrolysis of cellulose: noncomplexed cellulase systems. Biotechnol Bioeng..

[cit0035] Swatloski RP, Spear SK, Holbrey JD, Rogers RD (2002). Dissolution of cellulose with ionic liquid. J Am Chem Soc..

[cit0036] Li C, Zhao ZK (2007). Efficient acid-catalyzed hydrolysis of cellulose in ionic liquid. Adv Synthesis Catalysis..

[cit0037] Rinaldi R, Palkovits R, Schüth F (2008). Depolymerization of cellulose using solid catalysts in ionic liquids. Angew Chem Int Edition..

[cit0038] Su Y, Brown HM, Huang X, Zhou XD, Amonette JE, Zhang ZC (2009). Single-step conversion of cellulose to 5-hydroxymethylfurfural (HMF), a versatile platform chemical. Appl Catalysis A.

[cit0039] Su Y, Brown HM, Li G, Zhou D (2011). Accelerated cellulose depolymerization catalyzed by paired metal chlorides in ionic liquid solvent. Appl Catalysis A.

[cit0040] Hsu WH, Lee YY, Peng WH, Wu KC-W (2011). Cellulosic conversion in ionic liquids (ILs): Effects of H_2_O/cellulose molar ratios, temperatures, times, and different ILs on the production of monosaccharides and 5-hydroxymethylfurfural (HMF). Catalysis Today..

[cit0041] Li B, Filpponen I, Dimitris SA (2010). Acidolysis of wood in ionic liquids. Ind Eng Chem Res..

[cit0042] Tao F, Song H, Chou L (2011). Catalytic conversion of cellulose to chemicals in ionic liquid. Carbohydr Res..

[cit0043] Tao F, Song H, Chou L (2011). Hydrolysis of cellulose in SO_3_H-functionalized ionic liquids. Bioresour Technol..

[cit0044] Amarasekara AS, Wiredu B (2011). Degradation of cellulose in dilute aqueous solutions of acidic ionic liquid 1-(1-Propylsulfonic)-3-methylimidazolium chloride, and p-toluenesulfonic acid at moderate temperatures and pressures. Ind Eng Chem Res..

[cit0045] Liu Y, Xiao W, Xia S, Ma P (2013). SO_3_H-functionalized acidic ionic liquids as catalysts for the hydrolysis of cellulose. Carbohydr Polym.

[cit0046] Sasaki M, Kabyemela B, Malaluan R, Hirose S, Takeda N, Adschiri T, Arai K (1998). Cellulose hydrolysis in subcritical and supercritical water. J Supercrit Fluid..

[cit0047] Yu Y, Wu H (2010). Significant differences in the hydrolysis behavior of amorphous and crystalline portions within microcrystalline cellulose in hot-compressed water. Ind Eng Chem Res..

[cit0048] Wu Y, Fu Z, Yin D, Xu Q, Liu F, Lu C, Mao L (2010). Microwave-assisted hydrolysis of crystalline cellulose catalyzed by biomass char sulfonic acids. Green Chem..

[cit0049] Geboers J, Van de Vyver S, Carpentier K, de Blochouse K, Jacobs P, Sels B (2010). Efficient catalytic conversion of concentrated cellulose feeds to hexitols with heteropoly acids and Ru on carbon. Chem Commun..

[cit0050] Tan X, Deng W, Liu M, Zhang Q, Wang Y (2009). Carbon nanotube-supported gold nanoparticles as efficient catalysts for selective oxidation of cellobiose into gluconic acid in aqueous medium. Chem Commun..

